# Understanding Pathophysiological Complexity of Feline Hypertrophic Cardiomyopathy Using SWATH-MS Plasma Proteomics

**DOI:** 10.3390/ani16050781

**Published:** 2026-03-02

**Authors:** Halley Gora Ravuri, Andrea L. Daniels, Pawel Sadowski, Paul C. Mills

**Affiliations:** 1 School of Biomedical Sciences, The University of Queensland, St. Lucia, QLD 4072, Australia; 2 School of Veterinary Science, The University of Queensland, Gatton, QLD 4343, Australia; andrea.daniels@uqconnect.edu.au (A.L.D.); p.mills@uq.edu.au (P.C.M.); 3 Central Analytical Research Facility, Queensland University of Technology, Brisbane, QLD 4000, Australia; pawel.sadowski@qut.edu.au

**Keywords:** cats, data-independent acquisition, plasma proteomics, hypertrophic cardiomyopathy, complement activation, immunity

## Abstract

Establishing plasma biomarkers in the veterinary field has always been a challenge, due to a lack of significant understanding of pathophysiological attributes of disease. Advances in mass spectrometry-based proteomic techniques have improved plasma biomarker discovery in veterinary medicine. Feline hypertrophic cardiomyopathy is the most common cardiac disease in cats and has a complex and not fully elucidated pathophysiology. This study aimed to use SWATH-MS proteomics to identify novel plasma biomarkers for fHCM and to further elucidate disease pathogenesis. Plasma was collected from 20 cats, consisting of healthy controls (*n* = 10) and a HCM group *(n* = 10). Cats with fHCM, were diagnosed by echocardiography and disease statuses were determined by a veterinary cardiologist. Undepleted cat plasma samples were digested using FASP and quantitative analysis was performed using DIA-NN. A total of 40 plasma proteins were found to be dysregulated, primarily associated with innate and humoral responses, including complement C7 and C9 and properdin proteins. Other dysregulated proteins were involved in blood coagulation (fibrinogen, fibulin-1), lipid metabolism (apolipoproteins), and inflammation pathways (transthyretin and plasminogen). These findings provide possible biomarkers for fHCM, with the potential to detect disease before clinical signs become evident, which is a significant outcome for fHCM. These proteomic changes suggest critical pathways for earlier intervention and could potentially lead to more effective treatment outcomes. Furthermore, having significant similarity to human disease strengthens the case for using cats as a potential translational model for hHCM.

## Simple Summary

Feline hypertrophic cardiomyopathy (fHCM) is common cardiac disease, causing higher mortality rates in pet cats. This disease is often asymptomatic, and owners often only realise that their cat has a problem when overt clinical signs develop, which is often too late for any successful management. In this study, we compared the plasma proteome of cats clinically diagnosed with fHCM to healthy cats in an aim to identify possible proteomic dysregulation and understand this complex disease. Proteomic results revealed that there was significant involvement of protein dysregulation in various biological pathways. These dysregulated proteins may be useful in identifying disease onset at a much earlier stage, improving the possibility of treating and not just managing this insidious disease in cats and allowing for more effective monitoring of disease progression in affected cats. This study highlights the value of plasma proteomics in advancing our understanding of fHCM pathology and in identifying potential biomarkers. Another significant finding was that proteomic changes identified in feline HCM had similarity to human HCM and fosters the importance of cats as a human translatory model for studying cardiac diseases.

## 1. Introduction

Feline hypertrophic cardiomyopathy (fHCM) is the most common cardiac disease in cats, affecting approximately 15% of the population. fHCM has been noted to closely resemble the human disease (hHCM), including phenotype, clinical presentation, and histological changes, and thus makes an excellent translation animal model for HCM research [1,2,3,4]. Typically, cats are aged between 5 and 7 years old at diagnosis, and males are over-represented 3:1 compared to females [1,5]. Sarcomeric protein gene mutations are common in hHCM and have also been recognised in some cases of fHCM, with mutations in the MYHC7, MYPBP3 and ALMS1 genes identified in Maine Coons, Ragdoll and Sphynx breeds, respectively. Other cat breeds also show evidence of familial HCM, but many cases of feline HCM do not have a causative genetic aetiology associated with clinical disease [6,7,8,9,10,11]. The fHCM phenotype is associated with a heterogeneous clinical presentation and disease progression, and while some cats will remain sub-clinical, approximately 1/5 of cats will develop end-stage cardiomyopathy with severe outcomes [12], including congestive heart failure, feline arterial thromboembolism (FATE), and sudden cardiac death [13,14].

Cardiomyocytes have limited capacity for regeneration, and thus, following injury, repair consists of the clearance of necrotic tissue and the generation of fibrotic scar tissue [15,16]. The three phases of cardiac repair, inflammatory, proliferative and maturation, are tightly regulated to orchestrate optimal healing, with well-defined immune and stromal cell populations predominant during different phases of repair [17]. Persistent activation of the inflammatory response can lead to pathological myocardial remodelling [18]. Myocardial inflammation and immune system activation are key pathophysiological process that contribute to cardiac hypertrophy, fibrosis and dysfunction in human hypertrophic cardiomyopathy (hHCM) [13,14]. An initial chronic, low-grade inflammation, characterised by increased levels of pro-inflammatory cytokines and inflammatory cell infiltrates, and increased fibrosis often occurs [13]; however, the initiating factor/s that trigger this early and sustained low grade inflammation are currently unknown. Mechanical stress, potentially resulting from the disorganised sarcomere and cellular architecture, neuroendocrinological activation, mitochondrial oxidative stress and focal myocardial ischemia, have all been proposed [13,15]. Cats with fHCM have a similar early and ongoing pathophysiology, including leukocyte infiltration into the myocardium and alterations in the microvasculature [19,20,21,22].

The complement system is a key central component and amplifier of both the early innate inflammatory response and the adaptive immune system [23] and may play a role in the pathogenesis of fHCM [20]. Complement proteins have already been identified as having a role in hHCM [24,25,26,27] and may also be altered in fHCM [28,29]. The coagulation system may also be involved with both hHCM [30,31,32] and fHCM [33,34,35], with platelet activation and a hypercoagulable state identified, with increased frequency in cats with advanced disease or at an increased risk of a thrombotic event/FATE [34,36]. There is increasing evidence of an interaction between the two cascades [37,38,39], and this interaction may apply in the pathophysiology of HCM.

Recent advancements in biomarker discovery, especially using proteomic techniques, has enabled researchers to identify differentially abundant proteins and their signatures; categorise high-risk HCM subtypes in human cohort [40]; measure specific protein biomarkers in other heart diseases, including dilated, restrictive, and hypertrophic cardiomyopathies [25] and coronary artery disease [41]. Few studies have used proteomic techniques to identify potential plasma biomarkers in congestive heart failure [28] and study the efficacy of rapamycin to manage HCM in cats [29]. This study aimed to establish differential plasma proteomic profiles in clinically identified HCM positive cats, using sequential window acquisition of all theoretical fragment ion spectra (SWATH), one of the data-independent acquisition (DIA) approaches currently used for biomarker discovery in non-depleted plasma samples to explore pathophysiological alterations in veterinary species [42,43,44,45]. The findings in this explorative study would enable us to identify possible biomarkers and understand complex pathophysiology, which could be used for early detection of HCM condition in cats.

## 2. Materials and Methods

### 2.1. Animal Cohorts and Ethical Approval

This study was reviewed and approved by The University of Queensland Animal Ethics Committee (ethics approval number 2021/AE000457). Signed client consent forms for blood collection from the client owned cats and by the officer in charge of the UQ CSC colony of cats were obtained. Ten client-owned cats (6 males and 4 females) that were diagnosed with HCM of varying levels of severity were identified at initial diagnosis of HCM and ten HCM negative control cats (5 males and 5 females) were sourced from the UQ CSC colony cats. The breeds in the HCM cohort included domestic shorthaired (DSH), domestic longhaired (DLH), Ragdolls, Burmilla, British short haired and sphynx breeds, while only DSH and DLH were in the HCM negative control cohort. Detailed information about cats’ health, age, and clinical conditions are provided as Supplementary Files (S1).

### 2.2. Clinical Diagnosis of HCM in Cats

Hypertrophic cardiomyopathy positive cats were referred to a registered veterinary cardiologist by their regular attending veterinarian for cardiac assessment, usually following auscultation of a cardiac murmur at their local clinic, or as a pre-breeding assessment in genetically pre-disposed breeds. Hypertrophic cardiomyopathy negative cats were sourced from the Clinical Skills Centre (CSC) cat colony at the School of Veterinary Sciences, and had their echocardiograms were also performed by the specialist veterinary cardiologist to confirm their HCM negative status.

A routine physical examination was conducted, with an emphasis on cardiovascular parameters, e.g., capillary refill time, skin turgor, mucous membrane colour and femoral pulse rate/synchronicity, and any abnormalities observed were recorded. Pre-cordial palpation to assess any thrills and cardiac auscultation were then performed, as per established methods [46]. Briefly, systematic auscultation was carried out in a quiet room with the cat standing, or in a sternal position if uncooperative, to evaluate pulmonary sounds, heart rate, heart rhythm and assess any heart sounds. The locations of any murmurs auscultated were described as sternal or left or right para-sternal and graded on a scale of I to VI [47]. Previous medical history and complete blood count/blood biochemistry assessment was taken for each cat from the refereeing veterinarian/CSC facility manager, including the most recent assessments for co-morbid diseases including renal and thyroid diseases, diabetes, and systemic hypertension. Any cats that presented with renal disease (creatinine > 2.0 mg/dL), systemic hypertension (systolic blood pressure > 160 mmHg where able to be measured using the doppler or petMAP blood pressure monitors), hyperthyroidism (serum total T4 concentration > 4 µg/dL), diabetes mellitus, dehydration, congenital heart disease, cardiac neoplasia and any other systemic diseases or on any medication apart from routine parasite prophylaxis were excluded from the study. One cat (#5) that had just commenced frusemide treatment due to a diagnosis of stage 3 CHF was included in the study.

### 2.3. Echocardiography Analysis

Echocardiography was completed by a specialist veterinary cardiologist in all cats without sedation and all echocardiographic parameters were taken over three cardiac cycles and averaged. Concisely, the cat was lightly restrained in right-lateral recumbency on a custom-made foam echocardiography cushion with a cut-out piece to allow for probe placement on the thoracic wall. Echocardiography was performed with a Vivid™ iq Ultrasound system (GE Healthcare, Chicago, IL, USA) with a 12 MHz transducer. Routine two dimensional and M-mode echocardiography were completed for measurement of left ventricular and atrial dimensions in both systole and diastole, along with colour doppler interrogation of all cardiac valves and outflow tracks, with particular attention to the presence or absence of any systolic anterior motion of the mitral valve (SAM) causing dynamic left ventricular outflow tract obstruction (DLVOTO). The echocardiograms were recorded and analysed according to the American Society of Echocardiography and the Echocardiography Committee of the speciality of Cardiology, American College of Veterinary Internal Medicine. Diagnostic criteria for the diagnosis of HCM used the LVPWd and or/IVSd diameter of > 5.5 mm to potentially identify “pre-clinical” HCM cats [47]. Cats were then classified into the following groups: mild (IVSd and LVPWd thickness of 5.5–6.5 mm and LA/Ao < 1.5), moderate (IVSd and LVPWd thickness of 6.5–7.0 mm and LA/Ao < 1.8) and severe (IVSd and LVPWd thickness > 7.0 mm and LA/Ao > 1.8) [48].

### 2.4. Sample Collection and Storage

Blood was collected using a 22 G needle and approximately 3 mL of blood was collected via a sterile jugular venepuncture. This was immediately transferred into a 1.3 mL lithium heparin tube up to the fill line, and the sample was gently inverted 8–10 times to ensure adequate mixing and anticoagulation of the sample. The sample was then centrifuged at 2500× *g* for 10 min. The heparinised plasma supernatant was then removed using a disposable pipette without disturbing the underlying cell pellet and placed into a 1.5 mL Eppendorf tube and labelled with the date and the cat’s identifying details. This sample was then placed immediately on ice and transferred to a −80 °C freezer within an hour.

### 2.5. Proteomic Analysis of the Clinical Plasma Samples

Cat plasma samples were subjected to FASP digestion [49] using an automated JANUS G3 liquid handling platform (PerkinElmer, Waltham, MA, USA), enabled with an AcroPrep Advance 96-well filter plates (Pall Lab, 8075, Port Washington, NY, USA) and a vacuum manifold for both digestion and desalting [45]. Briefly, 40–50 µg of plasma protein prepared in 6M Urea-Tris buffer was loaded on to AcroPrep 30 kDa MWCO filter plate (Pall Lab, 8075, Port Washington, NY, USA) and pulled through using the vacuum. Subsequently, DTT-Urea Tris buffer (8 M urea and 25 mM DTT in 100 mM Tris-HCL at pH 8.5) was dispensed by the robot and samples were agitated for 1 hr at room temperature on a ThermoShake (Inheco, Planegg, Germany). Excess DTT was washed off using Urea-Tris buffer (8 M urea in 100 mM Tris-HCl at pH 8.5), and the protein samples were alkylated by adding IAM-Tris buffer (50 mM IAM, 8 M urea in 100 mM Tris-HCl, pH 8.5) and incubated for 20 min at room temperature in darkness. Alkylation was stopped by washing the samples with Urea-Tris buffer (8 M urea in 100 mM Tris-HCl, pH 8.5) twice to remove the IAM-Tris buffer, and then, the samples were washed with 100 mM Ammonium bicarbonate (AMBIC) twice. Trypsin (Promega, Cat. No. V5117, Madison, USA) digestion was performed on the samples (enzyme to protein ratio 1:50) while being agitated at 37 °C overnight. Digested tryptic peptides were desalted using solid phase extraction using an SCX membrane disk inserted into a StageTip [50]. Samples were then dried under vacuum and peptides resuspended using a solution containing 11 standard peptides (iRT Kit from Biognosys, Zürich, Switzerland) made up in 2% ACN in 0.1% FA and submitted for mass spectrometry analysis. The iRT peptides were used to calibrate RT and normalise SWATH-MS data for quantitative analysis.

### 2.6. Data-Independent Acquisition (DIA) Analysis of the Plasma Samples

For SWATH-MS analysis, all the digested plasma samples were analysed using a variable Q1 windows variant of SWATH on a TripleTOF 6600 quadrupole time-of-flight mass spectrometer (SCIEX) equipped with a DuoSpray Ion Source and coupled to an Eksigent ekspert nanoLC 400 System (Eksigent Technologies, Dublin, CA, USA) configured for microflow HPLC applications. The detailed instrument parameters were previously provided [51]. Briefly, the chromatographic separation involved trapping of peptides for 3 min at a flow rate of 10 μL per minute onto an Trajan Protecol trap (120 Å, 3 μm, 10 mm × 300 μm) followed by separation on an Eksigent ChromXP C18 3 μm 120 Å (3C18-CL-120, 3 μm, 120 Å, 0.3 × 150 mm) analytical column at a flow rate of 5 μL per minute maintained at 40 °C. Mobile phase A consisted of 0.1% FA in water and mobile phase B was made of 0.1% FA in ACN. Peptides were separated by a 68 min linear gradient of 3–25% mobile phase B followed by a 5 min linear gradient of 25–35% mobile phase B. High-resolution (30,000) TOF MS peptide ion scans were collected over a range of 350–1500 *m*/*z* for 50 ms and high- sensitivity TOF MS/MS fragment ion scans over a range of 100–1800 *m*/*z* over 100 variable Q1 windows (50 ms per window), resulting in a total duty cycle of 3.1 s. The parameters were optimized for doubly charged peptides.

### 2.7. Quantitative Data Processing

SWATH-MS data files are processed through Data-Independent Acquisition-Neural Network (DIA-NN) software (version 1.8) [52] for identification and quantitation of the peptides using *Felis Catus* FASTA, downloaded from the UniProt database (felis_catus, taxonomy 9685, with 51,849 entries). The quantitative information acquired after DIA-NN analysis was further processed using MS-STAT analysis in R programme (version 4.3.0). For initial peptide search and spectral library generation, the following parameters were enabled within the DIA-NN software (version 1.8): Library-free search enabled; Protease = Trypsin/P; N-terminal methionine excision enabled; Maximum number of missed cleavages set to 1; Min peptide length set to 7; Max peptide length set to 30; Min precursor *m*/*z* set to 300; Max precursor *m*/*z* set to 1250; Min precursor charge set to 2; Max precursor charge set to 4; and Cysteine carbamidomethylation enabled as a fixed modification. All the SWATH-MS data files were then analysed for quantitative information using the created library, with additional settings as follows: Protein inference = Genes; Neural network classifier = Double-pass mode; Cross-run normalisation = RT and signal dependent (experimental); Quantification strategy = Robust LC (high accuracy); and Library generation = Smart profiling. The report file (.tsv) (Supplementary File S2) obtained after DIA-NN analysis was then imported onto R-studio using the R package diann, and further quantitative analysis was performed at the peptide level by various R packages (MSstats version 4.8.2; DEqMS version 1.20; diann version 1.0.1; and dplyr version 1.1.2); all the peptides were filtered and normalized using following settings: data normalisation = log10 transform and equalise medians; filter by precursor q value ≤ 0.01; and filter by protein group q value ≤ 0.01. For data normalization and quality control, we used a two-step approach as implemented in the MSstats package. First, peptide-level intensities were log10-transformed to stabilize variance and improve the normality of the distribution. Then, median normalization was applied across runs to correct for systematic bias, such as differences in sample loading or instrument response. This procedure equalizes the median log-transformed intensities across all samples, ensuring comparability of abundance estimates. Additionally, peptides and proteins were filtered based on statistical confidence thresholds: precursor q-value ≤ 0.01 and protein group q-value ≤ 0.01, as estimated by DIA-NN. A 1% false-discover rate (FDR) with Benjamini–Hochberg multiple testing correction was applied to ensure stringent identification and quantitation of the proteins. The quantitative data information is obtained in excel format after MS-STATS analysis, and this file was used for further comparative analysis. Proteins with a *p*-value < 0.05 and with absolute log2 fold change of 0.5 were considered significantly dysregulated by comparing the control group with the HCM positive group. The quantitative data is then processed using VolcaNoseR [53] to visualise protein dysregulation as volcano plots. Differentially expressed proteins identified by SWATH-MS were subjected to gene ontology (GO) and Kyoto Encyclopedia of Genes and Genomes (KEGG) pathway annotation using ShinyGO (version 0.80) [54], and STRING (Version 11.5) (https://string-db.org) [55] was used for visualisation of protein–protein interactions and proteins associated with biological processes in this condition.

## 3. Results

### 3.1. Clinical Diagnosis of HCM Positive Cats

Based on electrocardiography analysis, cats were classified as mild (*n* = 4), moderate (*n* = 4) or severe (*n* = 2) HCM in this study. Individual cat echo data has been given in Table 1. Nine of the 10 HCM positive cats had SAM noted on echocardiography, graded as mild (*n* = 5), moderate (*n* = 3) or severe (*n* = 1), with 2/10 (#3 and #8) not having the LA/Ao, IVSs, IVPWs or %LVFS recorded, while cat #3 also displayed a regional focal apical HCM, so IVSd and LVPWd measurements were not recorded. Left atrial spontaneous echo contrast and evidence of left sided congestive heart-failure was noted in one cat (5). Forty percent of the cats (*n* = 4/10) were classified as mild fHCM, and the same number (*n* = 4/10) were classified as moderate fHCM per the previously detailed classification scheme [48]. Two of the cats were classified as having severe fHCM. All cats, except one, were classified as Stage B1 per ACVIM guidelines [10]. Left atrial spontaneous echo contrast, and evidence of stage 3 left-sided congestive heart-failure was noted in one cat, and it was thus staged as Stage C (Cat #5). Cat #2 presented with a focal apical hypertrophy, which is not captured in the standard echocardiographic measurements.

For a subset of cats that were presented for HCM screening due to genetic susceptibility (Cat #3 and Cat #8), some of the standard echocardiographic measurements were not completed by the specialist veterinary cardiologist. Although these measurements do allow an in-depth analysis of cardiac structure, the positive diagnosis of HCM was made using the above listed criteria, and they are not considered to have a significant impact on plasma proteomic signature of HCM positive cats. Clinical meta data of HCM positive cats is provided in a Supplementary File (S1).

Biochemistry data for most HCM positive cats were unremarkable, and combined with previous veterinary clinical history, comorbidities that could contribute to secondary fHCM were excluded. One cat (#2) had an elevated urea concentration of 16.1 mmol/L (5.0–15.0); however, creatinine was normal and the USG was 1.052 (1.035–1.060), and thus, renal disease could reasonably be excluded. One cat had (#5) a slightly elevated glucose concentration of 9.7 mmol/L (3.2–7.5) and considering the cat’s previous clinical history and demeanour during phlebotomy, this was considered a stress hyperglycaemia. Three cats did not have blood biochemistry recorded, as they were considered too young (4–10 months old) to be at risk from renal and thyroid disease or diabetes by the specialist veterinary cardiologist or the blood biochemistry had recently been completed by the referring veterinarian with no abnormalities detected and were therefore declined by the client (#3 and #8). Only one cat had a NT-proBNP performed by their referring veterinarian and this was consistent with the final diagnosis of fHCM.

### 3.2. Proteomic Analysis

In this study, MS-STAT analysis identified different numbers of proteins and corresponding peptides in both healthy and HCM positive samples at 1% FDR (Table 2). A total of 155 plasma proteins were quantified between groups (Supplementary File S3), and different proteins showed significant differences when compared between the groups. Volcano plots illustrate the upregulated and downregulated proteins between the groups. Proteins with a *p*-value < 0.05 and absolute log2 fold change of 0.5 were selected as differentially abundant proteins among groups for downstream pathways analysis and considered to be biologically relevant for the presented study cohort.

**Figure 1:**
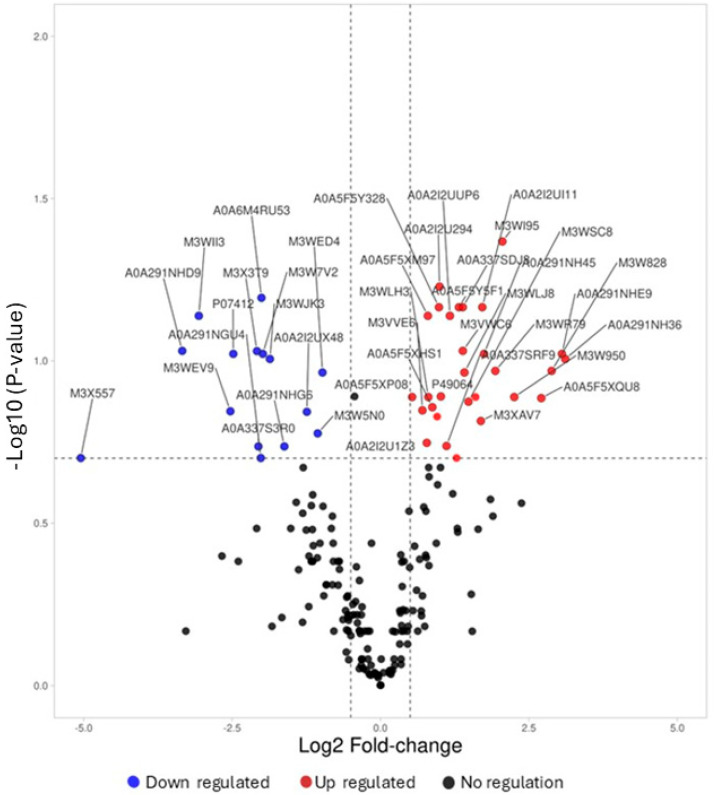
Volcano Plot showing dysregulated plasma proteins (*p*-value < 0.05) in HCM cats when compared to clinically healthy cats. The blue and red dots were used to indicate significantly down- and up-regulated proteins, respectively. The x-axis shows the log2 fold changes, where the y-axis shows the −log 10 *p*-value. The plot is defined with vertical dotted lines, which indicate the absolute fold change of 0.5 at a *p*-value < 0.05.

A total of 40 plasma proteins (with 14 downregulated and 26 upregulated proteins) were differentially expressed in HCM cats compared with the healthy control group of cats. A comparative list of all differentially abundant proteins with fold change is presented in Figure 1 and Table 3.

### 3.3. Gene Ontology and Protein Pathway Analysis

**Figure 2:**
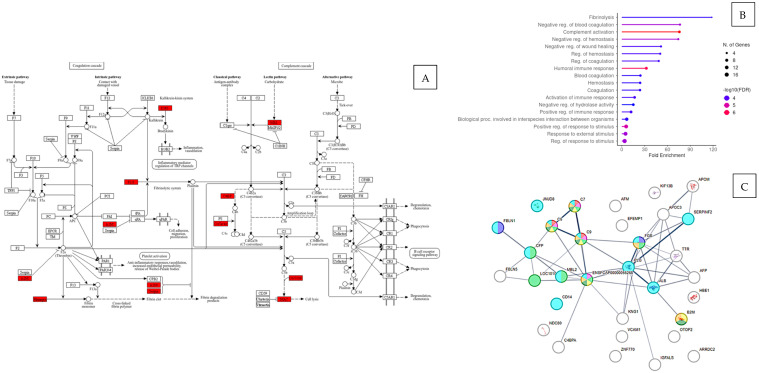
(**A**) KEGG pathway enrichment of dysregulated proteins (red boxes) involved in various lectin pathways and complement and coagulation cascades. (**B**) Enriched GO biological processes for dysregulated proteins showing involvement in blood coagulation, haemostasis processes, complement activation and activated immuno-inflammatory pathways. (**C**) Protein–protein interactions using the STRING software tool (version 11.5, 2024) identified significant interaction between significantly abundant proteins, suggesting a high complexity of systemic changes occurring in this condition.

KEGG pathway annotation (Figure 2A) and GO annotation analysis (Figure 2B) confirms that the dysregulated proteins were involved in different biological processes and pathophysiological pathways. STRING analysis (Figure 2C) showed significant clustering between these proteins, indicating a complex physiological interaction and their involvement in individual biological pathways (Table 4).

## 4. Discussion

Hypertrophic cardiomyopathy (HCM) is an inherited disease in cats and humans and is the most seen feline cardiac disease. A clinical diagnosis of HCM is made via echocardiography; however, by the time the disease is observable, pathological alterations in cardiac in structure and function are well reported [20]. Our study uniquely utilized samples from cats with actual, naturally occurring hypertrophic cardiomyopathy, providing direct insights into the disease’s pathophysiology in a clinical context. The identification of plasma proteomic markers for the disease could potentially aid early diagnosis and treatment strategies, along with disease progression and response to treatment monitoring. The plasma proteomic change in disease potentially offers insights into disease pathophysiology that could determine new treatment modalities. This study found dysregulation (Figure 1) in proteins associated with coagulation, inflammation, lipid metabolism, extracellular matrix (ECM) matrix proteins and the renin–angiotensin system (RAS). There is a complex interplay between the complement, coagulation and fibrinolytic systems (Figure 2A,C) in the circulatory inflammatory response in cardiovascular disease, and both coagulation and fibrinolytic activity have previously been noted in feline HCM [33,36,56].

The complement system is a key component of the innate immune response and in HCM positive cats there was significant upregulation of the following proteins: C9 (*p* = 0.0002, FC = 2.0533), complement factor properdin (*p* = 0.0179, FC = 2.2536), C7 (*p* = 0.0298, 1.6910 FC) and mannose binding lectin 1 (*p* = 0.0018; FC = 1.7146). Anaphylatoxin-like domain-containing protein (*p* = 0.0031, FC = 0.7981) and C6 (*p* = 0.0194, FC = 0.8061) were also upregulated using more relaxed cut-offs. Complement 9 protein (C9) is one of the five complement proteins (including C5b, C6, C7, C8 and C9) that comprise the circulating terminal complement complex (TCC), which represents the final events in the complement cascade. The upregulation of C9, C6 and C7 all indicate activation of the complement system in HCM cats, which agrees with previous studies where hHCM and myocardial infarction (analogous to the focal areas of myocardial ischemia proposed to play a role in the pathophysiology of HCM) also promote upregulation of plasma C6, C7 and C9 [57]. Since plasma and tissue C9 upregulate in myocardial infarction [58], and heart failure [25], focal areas of myocardial ischemia likely play a role in the pathophysiology of HCM, particularly the early phases of the disease. Furthermore, C9, C6 and C7 have been associated with CHF, and it is unclear if upregulation of these complement proteins is due to the underlying pathology or the subsequent CHF [59,60]. Interestingly, plasma levels of C9 are associated with thrombin generation [61] and may be contributing to the coagulation system dysregulation observed in the current study.

Properdin, a leucocyte-derived positive regulator of the complement system, was also significantly upregulated in HCM cats. Properdin acts by stabilising the alternative complement pathway convertases (C3bBbP and C5 convertases) and anchors it to activating surfaces, leading to increased C3 cleavage and contributing positively to complement activation [62,63]. Properdin is therefore an important amplifier of the three complement pathways and is evidence of alternative complement pathway activation in HCM cats [64]. Mature neutrophils release their internal stores of properdin upon stimulation with a variety of agonists, including C5a, tumour necrosis factor alpha (TNFα) and interleukin-8 (IL-8) [65]. Plasma properdin may also be derived from vascular endothelial cells in response to turbulent flow [66] and increases in the plasma concentration of properdin in fHCM may also be contributed to by turbulent blood flow, a known component of fHCM, and especially in those with hypertrophic obstructive cardiomyopathy (HOCM). A study identified that properdin was positively associated with endothelial dysfunction, low grade inflammation and cardiovascular events, including MI [67]. Interestingly, previous research has suggested that each of these three characteristics have been associated with HCM, including areas of focal micro myocardial ischemia [68,69].

Mannose binding lectin (MBL) is a circulating pattern recognition molecule that recognises a wide range of infectious agents, but also epitopes of apoptotic or necrotic cells [70]. It functions as an opsonin and forms complexes with MBL-associated serine proteases (MASPs) that activate the lectin complement pathway proteases and coagulation proteases and plays a central role in inflammation, coagulation and immunity [71]. Upregulated levels of MBL in the HCM cats gives an indication that the lectin complement pathway has been activated (Figure 2A), a finding that has also been observed in hHCM [24].

Anaphylatoxin-like domain-containing protein, although unspecified, its upregulation in the plasma of HCM cats represents the activation of the complement cascade. Both the anaphylatoxins C3a and C5a are potent inflammatory mediators and target a diverse range of both immune and non-immune cells [72]. The anaphylatoxins have been identified in plasma and implicated in the pathophysiology of hHCM [73]. Interestingly, in acute CHF, decreased levels of circulating C3a and C4a were observed and only C5a was increased [74]. Conversely, increased levels of C3a have also been observed in patients with CHF, and this was linked to other biomarkers of acute phase reactions, inflammation, endothelial cell activation and cellular stress responses [75]. Downregulation of sushi domain-containing protein (a C3b binding protein) further supports the hypothesis of dysregulation of the complement system in HCM cats.

Myocardial inflammation and immune system activation are key pathophysiological processes that contribute to cardiac hypertrophy, fibrosis and dysfunction in hHCM [18], but the initiating or trigger factor/s are currently unknown. Cats with fHCM have an early and ongoing pathophysiology, including leukocyte infiltration into the myocardium and alterations in the microvasculature [19,20,21]. This study is the first to identify that the complement system components and regulators, including the alternative and the lectin pathways are activated in fHCM and potentially contribute to the low grade-chronic inflammation identified in the early and continuing pathophysiology of HCM.

Feline aortic thromboembolism (FATE) is serious sequelae occurring in 11.3% of cats with HCM [76], reflecting hypercoagulability with altered secondary haemostatic biomarkers, including increased thrombin-antithrombin complexes, fibrin degradation products and D-dimers [36]. Importantly, 45% of cats with HCM have altered secondary haemostatic biomarkers [33] and systemic hypercoagulability is often present without concurrent CHF or FATE and may represent a risk factor for FATE [36]. Fibrinogen beta chain (*p* = 0.0036, FC = −3.0598) and plasminogen (*p* = 0.0064, FC = −2.0809) are associated with coagulation and fibrinolysis and were both downregulated in HCM cats. Fibrinogen is an acute phase protein, an important component of the coagulation cascade [77,78], and plays an important role in inflammation and atherogenesis [79]. As noted above, HCM is associated with coagulopathy, and thus the consumption and therefore downregulation of fibrinogen is unsurprising. A hyper-fibrino(geno)lysis consumes plasma fibrinogen in HCM cats, following reductions in plasma plasminogen as it is converted to active plasmin, the primary fibrinolysin [80]. Contrary to our findings, Stokol, et al. [36] identified that 37% of HCM positive cats had hyperfibrinogenemia and that median fibrinogen concentrations were elevated in HCM cats compared to healthy cats. However, another study reported that there is no difference in fibrinogen concentrations between control cats and cats with varying severities of HCM [34].

Systemic activation of the coagulation cascade (Figure 2A) can typically result in a complementary activation of the fibrinolytic cascade in an attempt to maintain homeostasis [81]. Both plasminogen and its active form plasmin play important physiological and pathological roles in fibrino(geno)lysis, haemostasis, degradation of ECM, cell migration, tissue re-modelling, angiogenesis and inflammation, all of which are present in the pathophysiology of HCM [82,83]. Degradation of ECM proteins assists immune cells, including macrophages and activated lymphocytes, to migrate into other tissues [83]. Plasminogen also inhibits complement by binding to C3, C3b, C5 and C4-binding protein [84], while plasmin conversely induces C3a and C5a with an efficiency similar to that of the complement convertases [85,86]. As noted earlier, the downregulation of plasminogen in HCM cats in this study likely reflects conversion to plasmin in response to hypercoagulation.

Serpin family F member 2 protein (also known as α 2 plasmin inhibitor; *p* = 0.0335, FC = −1.3090) is a major inhibitor of plasmin and plays a key role in inhibiting fibrinolysis [87]. It was significantly downregulated in cats with HCM and further supports evidence for coagulation dysregulation in fHCM. In contrast, kininogen 1 (*p* = 0.0006, FC = 0.9950), a pre-courser protein in the kallikrein–kinin system to high molecular weight kininogen (HWK), low molecular weight kininogen (LWK) and bradykinin that is cleaved from HWK by kallikrein [88,89], was upregulated in cats with HCM. HWK interacts with factor XII, factor XI and pre-kallikrein to initiate the intrinsic coagulation pathway and also inhibits the thrombin and plasmin induced aggregation of thrombocytes. The bradykinin that is released from HWK facilitates an increased vascular permeability, allowing leucocytes into the tissue, mediating inflammation [89]. HWK also downregulates endothelial cell proliferation and migration, inhibiting angiogenesis [90], potentially contributing to the coagulation dysregulation and decreased microvascular density observed in fHCM.

The concept of immunothrombosis has been proposed in the literature to reflect the interactions between the coagulation, complement and fibrinolytic systems [80,91]. Dysregulation of immunothrombosis during cardiovascular disease has been associated with systemic hyper-coagulopathy and the formation of thrombi [35]. Neutrophils are a significant participant in immunothrombosis, not only having roles in degranulation and phagocytosis, but also in releasing ‘neutrophil extra-cellular traps” (NETs) that have both immunological and pro-thrombotic properties. NETs have been shown to increase in cats with HCM, compared to controls [92], while NETs were triggered by C5a inducing acute lung injury [15] and suggests a similar process may be involved in the myocardium in HCM.

Fibulin-5 (*p* = 0.0206, FC = 2.7087) and fibulin-1 (*p* = 0.0385, FC = 1.1123) were both identified as being upregulated in cats with HCM, compared to controls. Fibulin-1 is an extra cellular matrix protein involved in tissue re-modelling [31] and has a soluble form in plasma where it interacts with fibrinogen and is incorporated into fibrin clots [93,94], which may be relevant in thrombus formation in cats with HCM. Fibulin-1 mRNA expression levels are increased in a murine model of DCM [95] and have been positively correlated with plasma NT-proBNP [96], suggesting a role in cardiovascular disease [97]. Furthermore, fibulin-5 contributes to the formation of elastogenic tissues, tissue homeostasis, and cardiovascular re-modelling and inhibits angiogenesis [31,98]. Cats (and humans) with HCM have been identified as having a reduced capillary density in the hypertrophic myocardium [22,99], not only due to inadequate compensatory growth but also a loss of capillaries during the pathogenesis of HCM [100]. The anti-angiogenic effects of fibulin-5 may contribute to this process in fHCM.

The renin–angiotensin system (RAS) is a hormonal cascade that has a central role in the maintenance of cardiovascular homeostasis via the regulation of blood pressure, salt and water balance. Dysregulation of RAS plays an important role in the progression of many cardiovascular diseases [101]. The classical RAS pathway involves the conversion of angiotensinogen to angiotensin I (AngI), by renin, and further conversion to the active peptide angiotensin II (AngII) by angiotensin converting enzyme (ACE). A second ACE gene has been discovered (termed ACE2) that converts AngI and AngII to Ang1–9 and Ang1–7 fragments, respectively. Although activation of the angiotensin II type 1 receptor by AngII early in cardiovascular disease plays an important compensatory role in maintaining homeostasis, long term activation of the RAS is maladaptive, promoting hypertension, fibrosis and left ventricular hypertrophy [102,103]. Historically, RAS analyte profiles have not been well described in cats with HCM, presumably due to the difficulties in accurately measuring angiotensin peptides, as they are rapidly degraded. Significant increases in plasma AngI, aldosterone, Ang1–7, PRA-s (a marker of plasma renin activity), and significant decrease in ACE-S (circulating marker of ACE activity) have been reported in HCM cats [104], although a different study reported no significant differences in any RAS peptide concentrations, including ACE-S activity and Ang1–7 proteins [105].

The current study found angiotensin-converting enzyme 2 (ACE2) (*p* = 0.0010, FC = −2.0041) and angiotensinogen (*p* = 0.0038, FC = 1.1697) dysregulated in HCM cats. Angiotensinogen is the only substrate for renin and thus is the sole precursor to all the angiotensin peptides in RAS [106]. At physiological levels, renin is not typically saturated by angiotensinogen, and thus even small increases in plasma angiotensinogen can result in significant increases in RAS activity [106]. The increases in plasma angiotensinogen in HCM cats supported RAS activation in fHCM and in hHCM [25], but expected elevations of AngII were not measured. However, AngII is rapidly either bound to its receptor or inactivated by angiotensinases and thus not expected to be represented in the dysregulated plasma proteins in HCM [107,108]. ACE2 is counter regulatory to the classical RAS because it converts AngI and AngII to Ang1–9 and Ang1–7, respectively, that are inactive at the type 1 angiotensin receptor. AT1–7 has been proposed to act via the MAS receptor to oppose RAS activation, thereby mediating vasodilation, antifibrotic effects, growth inhibition and anti-inflammatory effects [109,110]. ACE2 receptors are located densely in the vascular endothelium of the arterioles, arteries and the venules of the heart [111]. ACE is a membrane bound protease that can be shed to release the enzymically active extracellular domain [112] from the endothelial plasma membrane via a disintegrin and metalloproteases (ADAMs) 10 and 17, releasing it into the plasma [109]. Plasma ACE2 concentration has been observed to increase during heart failure and ischemic heart disease, conflicting with the reduction found in this study. Another study [113] identified increased cardiac tissue ACE2 in cats with HCM, isolated to the endothelium of the capillaries and medium sized arterioles, and sporadically in the tunica media of arterioles. The decreased plasma ACE2 in the current study may indicate a reduction in ADAM 10 and 17 activities, resulting in ACE2 being maintained on the endothelial plasma membrane and not shed into the plasma. However, the relationship between the biological roles of tissue and plasma ACE2 requires further investigation [114] but may provide insights into the currently controversial use of ACE inhibitors in fHCM. ACE2 has also been implicated in regulating thrombus formation and plays a role in coagulation haemostasis [115], potentially contributing to the dysregulation identified in fHCM.

There is likely altered lipid metabolism in cats with HCM [116]. The current study identified significant upregulation of apolipoprotein M (ApoM; *p* = 0.0119, FC = 2.8828) in cats with HCM, a protein associated with lipid metabolism. A similar increase in ApoM was reported in cats with congestive heart failure [28]. ApoM has effects on both lipoprotein and cholesterol metabolism [117], and its role in feline HCM requires further clarification. Apolipoprotein C-III (ApoC-III) was also upregulated in HCM cats in the current study. ApoC-III has a multidimensional influence on many pathophysiological processes, including TRL metabolism, promotion of inflammation, coagulation cascade and the progression of cardiovascular disease [118,119], but its potential role in fHCM needs to be further elucidated.

There were several proteins that were significantly dysregulated in the current study but cannot be sufficiently explained in the context of feline HCM. These include alpha fetoprotein (tumour associated foetal protein involved in oncogenic and ontogenic growth) [120]; transthyretin (a transport protein transporting thyroxine and retinol to the liver) [121]; and kinetochore protein NDC80 (a protein that directs microtubule dynamics required for chromosome segregation and spindle checkpoint activity) [122].

Various studies have also identified the pathophysiological similarities between both fHCM and human HCM condition and propose the use of cats as human translatory model for HCM [123,124]. The findings in this study were in agreement with previously published studies, where bioinformatic pathway analysis of HCM proteomics data have shown significant dysregulation in the Ras-MAPK pathway [40,125]; lipid metabolism [28]; extracellular matrix [126,127]; mitochondrial energetics [126]; inflammation [14,127,128,129]; innate immune system [28]; fibrosis [122,124]; and coagulation and complement pathways associated with human disease [27,40,60,127].

## 5. Limitations

This study had some limitations, including the small cohort numbers in both the HCM positive and control healthy groups and the differences in breed frequency between the two cohorts. As the study predominantly relied on availability of cats at veterinary clinics and owners’ consent to collect the blood from cats, the authors were only able to recruit 20 cats in this study. Due to this issue, there was a significant difference in ages between the two cohorts, with younger cats exhibiting a pro-inflammatory state compared to older cats that demonstrates a conversion to a pro-fibrotic state with older age [130,131]. For a subset of cats that were presented for HCM screening due to genetic susceptibility, some of the standard echocardiographic measurements were not completed by the specialist veterinary cardiologist. Although these measurements do allow an in-depth analysis of cardiac structure, the positive diagnosis of HCM was made using the above listed criteria and they are not considered to have a significant impact on plasma proteomic signature of HCM positive cats. The range of HCM severities recorded, with most cases being either mild or moderate HCM, were grouped for analysis so that trends between disease progression and correlations with clinical and echocardiographic features could not be discerned. Proteins associated with sarcomere mutations including MYH7 (β-myosin heavy chain), MYBPC3 (myosin-binding protein C), TNNT2 (troponin T), TNNI3 (troponin I), TPM1 (α-tropomyosin), MYL2/3 (myosin light chains), and ACTC1 (cardiac actin), were not identified in this plasma proteomic study. These proteins are locally released from cardiac myocyte and are present in very low concentrations in circulation [132], and presence of the dynamic range of highly abundant proteins in plasma further complicates the detection of small amounts of tissue proteins in the plasma [133], However, a recently published study had shown that detection and quantitation of troponins and mitochondrial proteins (especially extracellular matrix remodelling proteins) in plasma of sheep with myocardial infarction is possible when the peptide spectral library was generated using both plasma and cardiac tissue digest [134]. This could be another reason for lack of tissue specific proteins in our study, as detection of these proteins require cardiac tissue specific peptides incorporated into spectral library for proteomic analysis [135].

However, our study identified few extracellular matrix (ECM) proteins, which were reported to play a vital role in mitochondrial remodelling and fibrosis during myocardial infarction [134], including (i) fibronectin, an ECM protein responsible for cardiomyocyte hypertrophy and increased calcineurin and a nuclear factor of activated T-cell proliferation [136]; (ii) thrombospondin 1, a protein associated with inflammation and matrix remodelling and known to play a vital role in heart failure during myocardial infarction [137]; and (iii) cathelicidin, a protein known to play a vital role in myocardial ischemia activating cardiac inflammatory pathways [138]. A few other mitochondrial proteins, including mitochondrial calcium uptake 1 protein, 60 kDa mitochondrial heat shock protein, and mitochondrial ribosomal protein S28, were identified in this study. There are reports that the 60 kDa mitochondrial heat shock protein [139] and mitochondrial ribosomal protein S28 [140] may be associated with mitochondrial dysfunction in cardiac diseases, although these again were only detected in some cat samples and were therefore not statistically significant. This variability in the detection of specific proteins attests to the substantial phenotypic variability observed in cats with HCM. These dysregulated proteins identified in this study could be closely monitored in different stages of fHCM with clinical severity and investigations into correlations between plasma and cardiac tissue proteomics in future. While statistically significant proteomic differences were observed, we recognize that such findings may sometimes arise from technical or cohort-related variation, particularly with small sample sizes. To mitigate this, we did not rely solely on *p*-values but also considered absolute fold changes of proteins, as these provide a measure of effect size that is more reflective of biological relevance. Importantly, many of the proteins identified showed both significant statistical differences and consistent fold changes aligned with established HCM-related pathways, strengthening confidence that these findings may represent genuine disease-associated alterations. Nevertheless, these results should be interpreted as exploratory and warrant validation in larger cohorts in the future.

The authors would like to mention that lack of a completely annotated and comprehensive feline proteome database presented some difficulties when comparing to the highly annotated human proteome database. The proteome of veterinary species is not as highly annotated as human and or other model species and there is lot of redundancy and duplication of proteins and isoforms included with different accession numbers, which has always been an issue for proteomic analysis [141,142]. Another limitation is that proteomic data validation utilising Western blotting was not performed in this study due to a lack of availability of feline specific primary antibodies for most of the significantly altered proteins, which complicates the acquisition and has always been an issue with veterinary proteomics [143]. The results in this explorative study could potentially be validated by developing multiple reaction monitoring (MRM) assays for highly dysregulated proteins. However, this was out of the scope and timeline of the current study and could be achieved in the future for establishing targeted biomarker panels.

## 6. Conclusions

The present study demonstrates the feasibility and power of SWATH-MS proteomics to investigate the pathophysiological mechanisms associated with HCM in cats. From this proteomic investigation, it is evident that candidate plasma biomarkers were significantly associated with complement cascades, innate humoral responses, and coagulatory protein changes, suggesting that HCM is a complex condition involving systemic inflammation. The identified biomarkers have a potential to identify HCM before clinical disease is evident, with earlier therapeutic intervention (i.e., before structural changes occur) more likely to promote treatment successful rather than the current long-term management approach. Given the similarities between cat and human HCM conditions, cats could be used as a translational model for investigating age-related disease progression.

## Figures and Tables

**Table 1:** Echocardiography results of the HCM positive cats.

Cat #	HR	LA/Ao	IVSd (mm)	LVIDd (mm)	LVPWd (mm)	IVSs (mm)	LVIDs (mm)	LVPWs (mm)	%FS	HCM Severity	SAM	DLVOTO
1	234	1.19	6.25	16.53	6.11	9.03	7.08	9.44	57	mild	present	present, moderate, dynamic
2	174	1.16	3.94	19.79	3.59	5.9	9.84	5.09	50	mild, focal, apical hypertrophy	present	present, mild, dynamic
3	N/R	N/R	5.7	18.3	5	N/R	9.4	N/R	N/R	mild	N/R	N/R
4	146	1.6	7.52	12.6	6.82	8.44	7.05	7.86	44	moderate	present	present, moderate, dynamic
5	240	1.49	7.5	14.03	7.08	8.33	8.06	8.61	43	severe	present	present, mild, dynamic
6	240	1.34	7.18	11.46	6.13	8.1	4.86	7.64	58	moderate	present	present, mild, dynamic
7	168	1.31	7.29	11.69	7.41	8.45	5.44	8.1	53	severe	present	present, intermittent, mild, dynamic
8	N/R	N/R	6.7	20.7	6.8	N/R	9.2	N/R	55	moderate	present	present, mild, dynamic
9	200	1.48	6.02	15.63	4.4	6.25	7.06	5.79	55	mild	present	present, mild, dynamic
10	178	2.55	6.83	18.52	6.25	7.41	10.3	7.52	44	moderate	present	present, mild, dynamic

HR—heart rate; LA/Ao—left atrial appendage occlusion; IVSd—interventricular septal end diastole; LVIDd—left ventricular internal diameter end diastole; LVPWd—left ventricular posterior wall end diastole; IVSs—interventricular septal end systole; LVIDs—left ventricular internal diameter end systole; LVPWs—left ventricular posterior wall end systole; %FS—fractional shortening; SAM—systolic anterior motion of the mitral valve; N/R—not recorded; DLVOTO—dynamic left ventricular outflow tract obstruction.

**Table 2:** Summary of proteins and peptides identified in control (healthy) and HCM cats.

Group	Avg No. of Proteins Identified	Avg No. of Peptides Identified
Control (healthy)	218	1188
HCM positive	174	927

**Table 3:** List of differentially abundant proteins between the control and HCM positive groups, showing all dysregulated proteins with significant fold change.

Sl. No	Protein Name	Accession Code	*p*-Value	Fold Change	Regulation
1.	Alpha fetoprotein	M3X557	0.0472	−5.0482	Downregulated
2.	IgG constant region	A0A291NHG6	0.0057	−3.3388	Downregulated
3.	Fibrinogen beta chain	M3WII3	0.0036	−3.0598	Downregulated
4.	Transthyretin	M3WEV9	0.0256	−2.5288	Downregulated
5.	Haemoglobin subunit beta A/B	P07412	0.0080	−2.4789	Downregulated
6.	Plasminogen	M3X3T9	0.0064	−2.0809	Downregulated
7.	Sushi domain-containing protein	A0A337S3R0	0.0401	−2.0564	Downregulated
8.	IgH variable region	A0A291NGU4	0.0459	−2.0158	Downregulated
9.	Angiotensin-converting enzyme	A0A6M4RU53	0.0010	−2.0041	Downregulated
10.	Arrestin domain containing 2	M3W7V2	0.0078	−1.9810	Downregulated
11.	IgG constant region	A0A291NHE10	0.0406	−1.6192	Downregulated
12.	WD repeat domain 24	A0A2I2UX48	0.0264	−1.2396	Downregulated
13.	Serpin family F member 2	M3W5N0	0.0335	−1.0576	Downregulated
14.	Vascular cell adhesion molecule 1	M3WED4	0.0130	−0.9766	Downregulated
15.	Kinesin family member 13B	A0A5F5XP08	0.0185	0.5326	Upregulated
16.	EGF fibulin extracellular matrix protein 1	M3VVE6	0.0247	0.7063	Upregulated
17.	Afamin	A0A2I2U1Z3	0.0367	0.7805	Upregulated
18.	Anaphylatoxin containing protein	A0A5F5XM97	0.0031	0.7981	Upregulated
19.	Complement component C6	M3WLH3	0.0194	0.8061	Upregulated
20.	Zinc finger protein 770	A0A5F5XHS1	0.0234	0.8733	Upregulated
21.	Alpha-2-macroglobulin	A0A5F5Y328	0.0025	0.9845	Upregulated
22.	Kininogen 1	A0A2I2U294	0.0006	0.9950	Upregulated
23.	Albumin	P49064	0.0163	1.0177	Upregulated
24.	Fibulin-1	A0A337SRF9	0.0385	1.1123	Upregulated
25.	Angiotensinogen	A0A2I2UUP6	0.0038	1.1697	Upregulated
26.	IgG constant region	A0A291NHD9	0.0470	1.2814	Upregulated
27.	Insulin like growth factor binding protein	A0A337SDJ8	0.0025	1.3860	Upregulated
28.	Monocyte differentiation antigen CD14	M3VWC6	0.0059	1.3870	Upregulated
29.	Otopetrin 2	M3WLJ8	0.0131	1.4134	Upregulated
30.	Apolipoprotein C-III	M3WSC8	0.0218	1.4822	Upregulated
31.	Complement C7	M3XAV7	0.0298	1.6910	Upregulated
32.	Mannose binding lectin 1	A0A2I2UI11	0.0018	1.7146	Upregulated
33.	IgG lambda chain constant region	A0A291NH45	0.0082	1.7452	Upregulated
34.	Kinetochore protein NDC80	M3WR79	0.0118	1.9355	Upregulated
35.	Complement component C9	M3WI95	0.0002	2.0533	Upregulated
36.	Complement factor properdin	M3W950	0.0179	2.2536	Upregulated
37.	Fibulin-5	A0A5F5XQU8	0.0206	2.7087	Upregulated
38.	Apolipoprotein M	M3W828	0.0119	2.8828	Upregulated
39.	IgG constant region	A0A291NHE9	0.0085	3.0572	Upregulated
40.	IgG lambda chain constant region	A0A291NH36	0.0099	3.1130	Upregulated

**Table 4:** Enriched protein–protein interactions (*p* < 1.0 × 10^−16^ value) and reactome pathways of differentially abundant proteins to understand pathophysiological mechanisms in fHCM.

Colour Code	Pathway Description	Proteins Identified in the Network
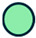	Humoral immune response	Complement factor properdin, complement 9, fibrinogen beta chain, complement 6, complement 7, C-type lectin domain-containing protein., anaphylatoxin-like domain-containing protein
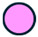	Complement activation, classical pathway	Complement C9, complement C6, complement C7, anaphylatoxin-like domain-containing protein
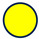	Lymphocyte mediated immunity	Beta-2-microglobulin, complements C9, C6, and C7, anaphylatoxin-like domain-containing protein
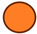	Adaptive immune response	Beta-2-microglobulin, fibrinogen beta chain, complement C9, complement C6, complement C7
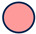	Cytolysis	Complement C9, complement C6, complement C7
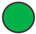	Adaptive immune response built from immunoglobulin superfamily domains	Beta-2-microglobulin, complement C9, complement C6, complement C7
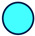	Response to stress	Monocyte differentiation antigen CD14, serpin family F member 2, complement factor properdin, complement C9, fibrinogen beta chain, C6, C7, serum albumin, C-type lectin domain-containing protein, WD repeat domain 24, fibulin 1, anaphylatoxin-like domain-containing protein
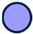	Blood coagulation, fibrin clot formation	fibrinogen, fibulin 1

## Data Availability

The mass spectrometry proteomics data files have been deposited into the ProteomeXchange Consortium via the PRIDE [144] partner repository with the dataset identifier PXD060893.

## Supplementary Materials

The following supporting information can be downloaded at: https://www.mdpi.com/article/10.3390/ani16050781/s1: clinical and echocardiography data of control (healthy) and HCM positive cats are provided as Supplementary File (S1); the quantitative data analysis report extracted from the DIA-NN analysis is available as Supplementary File (S2); and a list of total quantified proteins in the study is available as Supplementary File (S3).

## Abbreviations

The following abbreviations are used in this manuscript:

DIA	data-independent acquisition
DIA-NN	data-independent acquisition–neural networks
FASP	filter-aided sample preparation
FATE	feline arterial thromboembolism
fHCM	feline hypertrophic cardiomyopathy
hHCM	human hypertrophic cardiomyopathy
HCL	hydrochloric acid
IAM	iodoacetamide
MS/MS	tandem mass spectrometry
RT	retention time
SWATH-MS	sequential window acquisition of all theoretical fragment ion spectra and mass spectrometry
